# Evolutionary constraints impose multiple checkpoints that block mammalian sensory hair cell regeneration

**DOI:** 10.3389/fcell.2026.1835217

**Published:** 2026-05-20

**Authors:** Xiaoyi Zhang, Zhan Su, Dongmei Guan, Xinghong Liu, Hui Xie

**Affiliations:** 1 Chengdu University of Traditional Chinese Medicine, Chengdu, China; 2 Hospital of Chengdu University of Traditional Chinese Medicine, Chengdu, China

**Keywords:** auditory hair cells, microenvironment, progenitor cells, regeneration, signaling pathways

## Abstract

Irreversible damage to auditory hair cells is a primary etiology of sensorineural hearing loss. Across the vertebrate phylogenetic lineage, the regenerative capacity of these cells exhibits a marked decline: from robust regeneration in fish and amphibians, to functionally limited regeneration in birds, culminating in the permanent loss of this ability upon hair cell maturation in mammals. To elucidate the molecular logic underlying this evolutionary divergence and explore viable pathways for regeneration, this article employs a systematic cross-species comparative analysis. We propose that regenerating species share a highly conserved regenerative framework, whose most representative core components include: supporting cells as the key progenitor source, transcriptional reprogramming centered on genes such as Atonal homolog 1 (Atoh1), and a switch to a permissive microenvironmental state. The variation in regenerative capacity primarily stems from species-specific regulatory networks superimposed upon this core framework (e.g., the Forkhead box G1a (Foxg1a)/SRY-related HMG-box (SOX)/Sine oculis homeobox (Six) network in fish, the Fibroblast growth factor (FGF)-Extracellular signal-regulated kinase (ERK)-SOX2 cascade in birds), which fine-tune the efficiency and mode of regeneration. Based on this, we advance a “Multiple Checkpoints” hypothesis, positing that the acquisition of a regeneration-silent state in mammals is not due to the loss of the core framework. Instead, evolutionary processes have erected physiological barriers requiring coordinated overcoming at these key junctures, including: deep quiescence of progenitor cells, failure to initiate core reprogramming programs, and degeneration of the permissive microenvironment. This framework suggests that future therapeutic strategies aimed at achieving functional hearing restoration will likely require the concerted targeting of these multiple barriers. Through temporally precise interventions, microenvironmental remodeling, and combinatorial therapies, it may be possible to reactivate this evolutionarily dormant regenerative potential into a clinically viable new pathway.

## 1 Introduction

According to the World Health Organization (WHO), the global population with hearing loss has reached 1.5 billion and is projected to increase to 2.45 billion by 2050 (GBD, 2019 Hearing Loss Collaborators, 2021). Sensorineural hearing loss caused by irreversible damage to hair cells is prevalent, yet no existing intervention can replace or regenerate these cells. A clear phylogenetic gradient in regenerative capacity exists across vertebrates: efficient regeneration in fish and amphibians, functionally limited regeneration in birds, and near-complete loss in the mature mammalian cochlea. This evolutionary lock presents a fundamental biological puzzle, particularly given clinical observations that some patients with sudden hearing loss can show improvement after pharmacological intervention (Yeo et al., 2007; Li et al., 2025), suggesting latent regenerative potential. This raises a central question: is the loss of regenerative capacity in mammals due to the disappearance of a core regenerative module, or is its execution program systematically silenced?

A growing body of evidence indicates that the core genetic program driving hair cell differentiation is highly conserved across vertebrates, with Atoh1 retained and functional in mammals (Jahan et al., 2018). Recent interventions—including forced Atoh1 overexpression (Zhang et al., 2024), cell cycle inhibitor removal (Shu et al., 2019), and microenvironmental modulation (Liu et al., 2024) — have successfully induced hair cell-like cells and shown limited functional improvement, suggesting that the regenerative machinery is present but silenced. Recently, a study published in The Lancet reported successful treatment of hereditary deafness via AAV-mediated Otoferlin (OTOF) gene delivery (Lv et al., 2024), demonstrating the feasibility of precise inner ear intervention. Building on these insights and cross-species comparisons, this article synthesizes evidence to test the hypothesis that mammalian hair cell regeneration failure results not from the loss of core reprogramming programs, but from the evolutionary accretion of multi-layered inhibitory checkpoints that cooperatively lock the regenerative pathway.

## 2 The evolutionary framework of auditory hair cell regeneration: from conserved core modules to species-specific regulation

### 2.1 Conserved core modules of vertebrate hair cell regeneration

A comparative analysis of hair cell regeneration capabilities among fish, amphibians, and birds reveals that, despite variations in regenerative efficiency and regulatory details, a common logical framework underpinned by three key components exists. This framework does not imply that all species operate in an identical manner but rather suggests that successful auditory hair cell regeneration appears to depend on the coordinated activation of these three components, ultimately manifested in some form across species.

#### 2.1.1 Supporting cells as the source of regenerative progenitors

Although the specific triggering conditions and cellular subtypes for auditory hair cell activation vary, extensive research has confirmed that in regenerative-competent non-mammalian species (such as birds and fish), supporting cells indeed serve as the primary and direct source for hair cell regeneration. In birds, supporting cells generate new hair cells either through mitosis (primarily producing tall hair cells) or via direct transdifferentiation (primarily producing short hair cells) (Bermingham-McDonogh and Rubel, 2003; Janesick et al., 2022; Miranda Portillo et al., 2025; Ryals and Rubel, 1988; Walshe et al., 2003). In the zebrafish lateral line, definitive lineage tracing has confirmed that supporting cells are direct precursors of hair cells (Romero-Carvajal et al., 2015; Lush et al., 2019; Thomas and Raible, 2019). In amphibians, even under conditions of inhibited cell division, the predominant role of supporting cell transdifferentiation has been clearly demonstrated (Taylor and Forge, 2005). Similarly, in the zebrafish inner ear, transdifferentiation has been shown to be a major mechanism of hair cell regeneration following ototoxic injury (Beaulieu et al., 2024), further supporting the notion that supporting cell transdifferentiation is a widely conserved regenerative strategy across vertebrates. Therefore, “supporting cells reacquiring plasticity” represents a fundamental common starting point for regenerative events across species.

#### 2.1.2 The core hub atoh1 determines hair cell fate

The transcription factor Atoh1 is necessary and sufficient for hair cell differentiation across all vertebrate species examined. Its expression is tightly correlated with nascent hair cells in regenerating avian and zebrafish epithelia, and loss of Atoh1 function severely impairs regeneration (Jimenez et al., 2022; Ryals and Rubel, 1988). In mammals, Atoh1 is not only retained but also exhibits conserved developmental function (Jahan et al., 2018). Its activity is modulated by multiple layers of regulation: Notch signalling represses Atoh1 transcription, and its inhibition releases Atoh1 to drive hair cell formation (Samarajeewa et al., 2019); SOX2 both promotes Atoh1 transcription and targets it for proteasomal degradation via Huwe1, finetuning Atoh1 protein levels (Cheng et al., 2025); and the Atoh1 transcriptional programme is complemented by co-factors such as Pou4f3, Gfi1 and the miR-183 family, which together orchestrate the full hair cell gene network (Chen et al., 2018; Ebeid et al., 2017; Zhang et al., 2024). The precise timing and dosage of Atoh1 expression are critical: insufficient or truncated expression leads to immature stereocilia bundles and subsequent hair cell death (Pan et al., 2012).

#### 2.1.3 Transition of the microenvironment from static support to regeneration-permissiveness

Successful regeneration requires a coordinated shift in the local signalling microenvironment from homeostatic maintenance to regeneration-permissive conditions. Early electrophysiological and structural studies in birds and amphibians established the fundamental operating principles of hair cells and the tuning properties of the auditory epithelium (Ashmore, 1983; Hillery and Narins, 1984; Hudspeth, 1985; Lewis et al., 1982; Oberholtzer et al., 1988; Tilney and Saunders, 1983), providing the physiological baseline against which regenerative changes are measured. In zebrafish, hair cell ablation triggers rapid downregulation of Notch signalling and activation of Wnt signalling in supporting cells, both of which are required for regeneration (Bell et al., 2024; Lush et al., 2019; Mizutari et al., 2013; Wang et al., 2023; Xu and Yang, 2021). In birds, activation of cAMP signalling by forskolin upregulates a broad programme of cell cycle and developmental genes, with miR-181a identified as a key pro-proliferative microRNA (Frucht et al., 2010). In mammals, experimental activation of Myc, YAP, ERBB2 or inhibition of Hippo signalling can provoke supporting cell proliferation, demonstrating that permissive signals can be artificially supplied (Lu et al., 2022; Shu et al., 2019; Zhang et al., 2018). Extracellular matrix composition also shifts during regeneration, and decellularized ECM scaffolds from regenerative species promote hair cell differentiation *in vitro* (Arkenberg et al., 2025; Killick et al., 1995; Zhang J. et al., 2023). These observations converge on a conserved principle: the microenvironment must transition from a “non-permissive” to a “permissive” state for regeneration to proceed, although the dominant signals vary across species.

### 2.2 Key determinants of regenerative efficiency: species-specific regulatory modifications

The regeneration of vertebrate auditory hair cells relies on a core module comprising supporting cells as the source and conserved signaling pathways (e.g., Wnt/Notch, Hippo/YAP). However, cross-species comparisons reveal that regenerative capacity presents as an “evolutionary attenuation spectrum.” This variation is primarily attributed to species-specific regulatory modification networks superimposed upon the core module. These modifications broadly involve systemic-level processes such as immune responses (e.g., macrophage function (Barthelaix et al., 2025), endocrine regulation, and metabolic homeostasis maintenance (Losner et al., 2021). They either enhance or finely constrain the initiation and progression of regeneration, ultimately collectively shaping the continuous phenotypic spectrum from the highly efficient and complete regeneration in fish to the functionally limited regeneration in birds.

#### 2.2.1 Fish: enhanced networks and systemic plasticity underlying high-efficiency regeneration

Fish models, such as zebrafish, hold distinct advantages for studying auditory hair cell regeneration patterns. Their near-complete regenerative capacity stems from a robust, multi-layered enhancement system.

In fish, supporting cells not only possess the plasticity to transform into hair cells but their regenerative program is also powerfully driven by an efficient core transcriptional network. Studies have found that transcription factors of the Sox and Six families are rapidly activated in supporting cells post-injury, creating a molecular environment conducive to transdifferentiation (Jimenez et al., 2022). The key gene Foxg1a plays multiple roles, not only regulating developmental tempo but also actively driving cell fate transitions during regeneration (Bell et al., 2024). The deep involvement of such multifunctional genes is a hallmark of highly efficient regeneration in fish. Furthermore, fish models exhibit dynamic and precise regulation of Notch signaling. Specifically, Notch signaling in fish regeneration is not merely an inhibitory “brake” but functions as a dynamic, bidirectional regulatory tool. During the initial phase of regeneration, timely downregulation of Notch signaling permits supporting cells to initiate the transformation program (Liu et al., 2021; Wang et al., 2023). Subsequent precise re-establishment of this signaling may then serve to control the appropriate number and arrangement of regenerated hair cells, preventing over-proliferation. This fine-tuned control over a conserved pathway is a hallmark of efficient regeneration. At the metabolic and systemic levels, the regenerative capacity of fish is supported by system-wide mechanisms. For example, glutamine synthetase (GS) directly participates in and ensures hair cell differentiation and maturation by providing the metabolite glutamine (Zhao et al., 2024). Additionally, the lateral line system, being exposed on the body surface, likely provides a more readily established permissive microenvironment (e.g., richer blood supply, more flexible immune responses), offering ideal conditions for rapid regeneration. Notably,the functional enhancement of conserved genes provides a basis for efficient hair cell regeneration in fish. Genes that primarily participate in hearing in mammals, in addition to their auditory functions in fish, are more directly involved in hair cell development and maintenance, thereby becoming highly associated with regenerative capacity. For example, genes such as ATP-Binding Cassette Subfamily B Member 6 (Abcb6), Transmembrane Channel-like1/2 (TMC1/2), and Small Muscle Protein, X-linked (Smpx) are primarily associated with hearing loss in mammals (Baril et al., 2024; Ghilardi et al., 2021; Wang et al., 2025); however, in fish, loss of their function directly causes defects in hair cell development or maintenance (Smith et al., 2020). This suggests that fish have more tightly integrated these conserved genes into the regulatory pathways of hair cell regeneration, enabling them to assume more critical functions during the regenerative process.

In summary, no single gene has been identified that can explain the robust hair cell regenerative advantage observed in fish. Instead, it benefits from an enhanced network that seamlessly integrates core transcriptional drivers, dynamic signal regulation, metabolic coordination, and a favorable anatomical microenvironment. This integrated system allows the core regenerative module to operate with near-optimal efficiency.

#### 2.2.2 Birds: fine-tuned regulation and pathway specialization in regeneration

Although the regenerative capacity of avian auditory hair cells is less robust than that of fish, the strong and near-complete regenerative ability of birds makes them a critical bridge for studying mammalian auditory hair cell regeneration. Building upon the core module, avian regenerative mechanisms possess a finely tuned regulatory and specialized system, yet they also face more constraints when initiating regeneration.

First, avian hair cell regeneration strictly relies on the FGF→ERK→SOX2 signaling axis, and blocking this pathway completely abolishes their regenerative capacity. Although this core signaling pathway also plays a role in fish hair cell regeneration, birds exhibit a highly specific and indispensable dependence on this cascade, whereas fish possess more extensive signaling compensation and regulatory redundancy (Benkafadar et al., 2024). In other words, this stringent signaling dependence ensures the precision of regeneration but also creates potential regulatory bottlenecks, making the avian regenerative program more conditionally restricted compared to that of fish. Second, supporting cells within the avian basilar papilla exhibit clear spatial heterogeneity: neural-side supporting cells tend to regenerate tall hair cells via mitosis, whereas abneural-side supporting cells predominantly generate short hair cells through direct transdifferentiation (Bermingham-McDonogh and Rubel, 2003; Walshe et al., 2003). This compartmentalized and stereotyped regenerative mode reduces the overall responsiveness flexibility of supporting cells. Given that mammalian supporting cells exhibit even higher heterogeneity yet lack regenerative capacity, heterogeneity *per se* cannot determine regenerative potential. The relatively weak regenerative capacity in birds is more likely attributable to the functional specialization and restricted plasticity associated with their heterogeneity, rather than the presence or absence of heterogeneity *per se*. This spatial position-dependent specialization of cell subtypes and pathway selection represents a key species-specific regulatory modification in birds, enabling cell-type specificity in regeneration while shaping its unique regenerative dynamics. Moreover, in the regulation of hair cell precursor migration and maturation, EDNRB signaling plays an irreplaceable role in avian regeneration, and pharmacological inhibition of this pathway leads to impaired migration and delayed maturation of auditory hair cell precursors (Takeuchi et al., 2025). This suggests that avian regeneration requires an additional step to coordinate spatial cell movement—a process that may be simplified or absent in the structurally simpler and faster-regenerating zebrafish lateral line system. Finally, unlike in fish, regenerated hair cells in birds establish a distinctive “inside-out” synaptic pattern (Sato et al., 2024), and their neural reintegration mechanism represents a specialized repair mode adapted to the post-injury environment rather than a complete recapitulation of the developmental program.

In summary, avian regeneration is characterized by refined regulation, pathway specialization, and condition dependence. Importantly, these specialized modifications do not compromise the completeness of avian hair cell regeneration; rather, they shape a unique regenerative phenotype through cellular subtype specialization, stringent signaling dependence, and complex maturation steps, thereby illustrating the evolutionary diversity of vertebrate auditory hair cell regeneration mechanisms.

#### 2.2.3 Amphibians: unique regenerative phenotype in evolutionary transition

As a key taxonomic group transitioning from aquatic to terrestrial life (Wang et al., 2024; Warchol, 2011), amphibians provide important reference value for studying the evolution of auditory hair cell regeneration. Compared with fish and birds, amphibians (e.g., salamanders, *Xenopus*) exhibit a unique regenerative strategy characterized by multiple features rather than falling simply into a fixed capacity gradient. Similar to fish, amphibian supporting cells can generate new hair cells through direct transdifferentiation after hair cell loss, a process that can still occur even when mitosis is inhibited (Taylor and Forge, 2005). Concurrently, multiple studies have confirmed that amphibian supporting cells can also participate in regeneration via mitosis, showing clear similarities to the regenerative pattern observed in birds (Baird et al., 1996; Jones and Corwin, 1996). Thus, their regeneration does not rely on a single mechanism but represents a composite mode in which transdifferentiation and proliferation coexist. Notably, the proliferative aspect of hair cell regeneration in the zebrafish lateral line has been proposed as a specialized event in which terminal cell division participates in hair cell polarity establishment (Lush et al., 2019). In contrast, the proliferative regeneration observed in amphibians does not appear to exhibit such obvious functional specialization, further suggesting that the amphibian regenerative mechanism may represent a more basal, less specialized state during the transition from aquatic to terrestrial vertebrates (Alibardi, 2023; Wang et al., 2024). In terms of regenerative efficiency and dynamics, amphibians lack the rapid, large-scale proliferative regeneration seen in the zebrafish lateral line system, and their overall regenerative response more closely resembles the regulatory characteristics of terrestrial vertebrates. Single-cell transcriptomic comparisons have revealed that the amphibian inner ear retains some fish-like regeneration-associated gene expression signatures while also exhibiting downregulation of certain signaling pathways, presenting molecular features transitional toward more derived vertebrates. At the cellular level, amphibian supporting cells display marked morphological plasticity following extensive hair cell loss, expanding their apical surface area to form temporary filling structures (Taylor and Forge, 2005)—a response that shows evolutionary connections to and differences from the permanent fibrotic scarring that ultimately forms in mammals. Notably, the inner ear neuronal cell types are highly conserved between *Xenopus* and mice.

In summary, amphibians may represent an evolutionary basal state of regenerative capacity following the transition of tetrapods to land. They have retained the ancestral core regulatory modules for regeneration but, unlike fish, did not continuously enhance regeneration-associated regulatory networks during evolution, nor did they develop the finely tuned, highly specialized repair regulatory patterns observed in birds. Consequently, their regenerative efficiency lies between the two. Given that amphibians evolved from aquatic fish ancestors, their relatively limited regenerative enhancement networks are more likely the result of secondary weakening or partial loss rather than a failure to acquire such regulatory mechanisms. Overall, the evolution of regenerative capacity in vertebrates does not follow a simple linear decline from fish to mammals. Instead, during the adaptive transition from aquatic to terrestrial life, accompanied by changes in ecological demands, developmental programs, and immune environments, lineage-specific remodeling, adjustment, and even loss of regenerative capacity have occurred. This positions amphibians as a key evolutionary node for understanding how regenerative capacity in tetrapods has been modified or progressively lost.

### 2.3 Regenerative barriers in mammals: lockdown of the core module by multiple checkpoints

**FIGURE 1:**
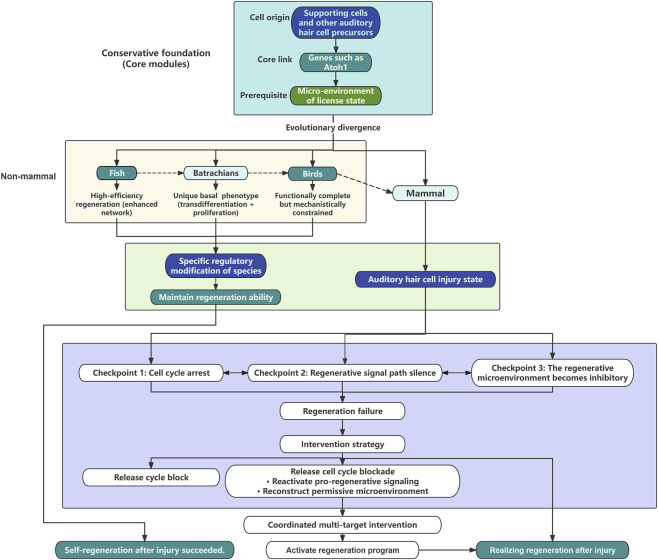
Schematic flowchart of the evolutionary framework and the checkpoint hypothesis for auditory hair cell regeneration in vertebrates. The diagram starts with a conserved core module: supporting cells as progenitors, Atoh1 as the master regulator, and a permissive microenvironment. It then shows evolutionary divergence in non-mammalian vertebrates: fish exhibit high-efficiency regeneration via enhanced networks; amphibians (batrachians) display a unique basal phenotype where transdifferentiation and proliferation coexist; birds show functionally complete but mechanistically constrained regeneration (e.g., stringent FGF-ERK-SOX2 dependence). Mammalian regenerative failure arises from three synergistic checkpoints that form a self-reinforcing inhibitory network: cell cycle arrest and epigenetic locking, silencing of pro-regenerative signaling, and deterioration of the permissive microenvironment. Double-headed arrows indicate bidirectional coupling among checkpoints. The flowchart concludes that successful regeneration requires coordinated multi-target intervention addressing all three checkpoints: releasing cell cycle blockade, reactivating pro-regenerative signaling pathways, and reconstructing a permissive microenvironment. Atoh1 (Atonal homolog 1) is the master regulatory factor determining hair cell fate.

Compared to fish, amphibians, and birds, the mature cochlea of mammals—including humans—exhibits an almost complete loss of regenerative capacity. This loss is not due to the absence of regeneration-associated genes (e.g., Atoh1), which remain present and functionally conserved in mammals (Koehler et al., 2017), but rather results from the evolution of an exceptionally robust multi-layered inhibitory system. We propose that during the evolution from non-mammals to mammals, one or more layers of increasingly robust physiological inhibitory barriers have been superimposed upon each key permissive regulatory node of the regeneration process. These “checkpoints” (as shown in Figure 1) primarily include: (1) the deep quiescence and epigenetic locking of progenitor cells (supporting cells); (2) the silencing of endogenous pro-regenerative signaling pathways; and (3) the degradation of the supportive microenvironment and associated metabolic challenges. Crucially, these elements do not operate in isolation but function synergistically, collectively and systematically locking the once-active regenerative program into a state of deep quiescence. This “Multiple Checkpoints” hypothesis provides a systematic explanation for the regenerative blockade in the mammalian inner ear.

#### 2.3.1 Checkpoint one: deep quiescence and epigenetic locking of regenerative progenitor cells

In regenerative models, dedifferentiation and reactivation of supporting cells mark the onset of regeneration. However, in mammals, cochlear supporting cells enter a deep and stable quiescent state early after birth.

Unlike in birds, where supporting cells rapidly enter S-phase after injury (Portillo et al., 2025), mammalian supporting cells exhibit sustained high expression and form a stable network of core cell cycle inhibitors, including p27Kip1, p19Ink4d, and the retinoblastoma (RB) protein family (Groves et al., 2013). These factors potently inhibit the activity of cyclin-dependent kinases (CDKs), constructing a nearly insurmountable G1/S barrier that permanently arrests supporting cells in the G0/G1 phase. Furthermore, while Notch signaling demonstrates flexible dynamic downregulation during regeneration in fish and birds (Mizutari et al., 2013; Xu and Yang, 2021), its persistently high activity in the adult mammalian cochlea plays a distinctly different role. Notch signaling may partially suppress hair cell regeneration in the adult mammalian inner ear, but its inhibitory effect is strongly age-dependent and modulated by broader epigenetic states, rather than acting as a standalone repressive mechanism. Indeed, the responsiveness of cochlear supporting cells to Notch inhibition declines dramatically with postnatal age, becoming minimal in adulthood (Maass et al., 2015). Importantly, epigenetic remodeling precedes the loss of Notch-dependent regenerative competence, indicating that epigenetic restriction is an upstream event that limits regenerative potential (Tao et al., 2021). Therefore, rather than Notch serving as a discrete “brake,” mammalian regenerative incompetence likely arises from a combined regulatory network involving epigenetic silencing, diminished chromatin accessibility, and altered Notch signaling. Consistent with this, recent work has highlighted that widespread losses in chromatin accessibility represent a core evolutionary barrier that distinguishes non-regenerating mammals from robust regenerators such as fish and birds (Shi et al., 2024). Sustained Notch signaling effectively blocks attempts by supporting cells to transdifferentiate toward a hair cell fate by inhibiting pro-differentiation factors such as Atoh1. Additionally, chromatin accessibility and histone modification profiles are crucial determinants of cellular plasticity. Studies show that, compared to birds, the chromatin of adult mammalian cochlear supporting cells is in a more “closed” state, with promoter regions of regeneration-associated genes occupied by repressive histone marks (e.g., H3K9me3, H3K27me3) (Buswinka et al., 2024; Wu et al., 2023). This epigenetic “locking” renders the initiation of the regenerative program extremely difficult, even in the presence of appropriate transcription factors.

#### 2.3.2 Checkpoint two: silencing of endogenous pro-regenerative signaling pathways

In highly efficient regeneration models, injury rapidly triggers a permissive signaling microenvironment. The mammalian cochlea, in contrast, critically lacks this endogenous initiation capacity.

On one hand, the Wnt/β-catenin and FGF signaling pathways, which are indispensable for avian regeneration, exhibit extremely low endogenous activity in the adult mammalian cochlea (Tang et al., 2022). Although these pathways are highly active during development, driving the proliferation of sensory progenitors, their ligand expression declines substantially after birth, and receptor sensitivity may also be altered. While exogenous activation of these pathways can stimulate supporting cell proliferation to some extent, demonstrating that their downstream machinery remains intact, the endogenous “ignition” signal is essentially absent. On the other hand, the absence of such permissive signals (e.g., Wnt/β-catenin, FGF) in mammals is accompanied by the sustained presence of inhibitory signals. For instance, signaling pathways such as Notch and the transforming growth factor-beta (TGF-β) superfamily, which are generally growth-inhibitory, likely play roles in suppressing regeneration in the adult cochlea. Moreover, structural maturation of the cochlear epithelium itself contributes to the loss of regenerative capacity (Burns and Corwin, 2014). Demonstrated that supporting cells in the mammalian cochlea progressively lose their proliferative capacity as the epithelium matures, and this decline correlates with the establishment of cell-cell contacts and apical junctional complexes rather than with an intrinsic cell cycle arrest alone. This finding suggests that the physical architecture of the mature mammalian cochlea actively suppresses the responsiveness of supporting cells to pro-regenerative signals. Consistent with the discussion above, these signals do not function as autonomous checkpoints but rather as components of a series of coupled systems that integrate epigenetic constraints, age-related limitations, and structural maturation of the sensory epithelium.

Finally, the composition of the extracellular matrix (ECM) also undergoes changes, potentially becoming enriched with molecules that inhibit cell migration and proliferation (e.g., certain chondroitin sulfate proteoglycans). This results in a matrix environment that is “non-permissive” both physically and chemically (Arkenberg et al., 2025; Zhang J. et al., 2023).

#### 2.3.3 Checkpoint three: severe degeneration of the supportive microenvironment and metabolic challenges

A microenvironment permissive for regeneration requires adequate blood supply, nutrients, and metabolic support. However, the highly specialized structure of the mammalian cochlea poses unique obstacles in this regard.

First, the mammalian cochlea exhibits a spiral structure with tight intercellular junctions, rendering its internal vascular systems, such as the stria vascularis and spiral ligament, relatively enclosed and distinctly segregated from the surrounding tissues. This highly isolated microenvironment, on one hand, satisfies the high metabolic demands and maintenance of internal homeostasis of the cochlea, but on the other hand, limits effective nutrient supply, cell migration, and delivery of repair signals following injury. The enclosed vascular architecture, combined with robust metabolic demands, collectively results in insufficient nutrient and oxygen supply to the damaged area, poor clearance of inflammation, and restricted diffusion of regenerative signals. These factors subsequently inhibit supporting cell reprogramming, proliferation, and *de novo* hair cell formation, ultimately leading to a markedly restricted regenerative capacity in the mammalian cochlea. Indeed, pathological conditions of the cochlear vasculature (e.g., deficiency of Norrin protein) directly lead to progressive hair cell loss (Patel et al., 2024), underscoring the fundamental role of an intact vascular microenvironment in hair cell survival. Concurrently, the expression levels of neurotrophic factors (e.g., brain-derived neurotrophic factor (BDNF) and neurotrophin-3 (NT-3)) in the adult cochlea may be insufficient to support large-scale *de novo* neuronal synaptic reconstruction. Second, hair cells across vertebrates, including both mammals and zebrafish, are susceptible to oxidative stress and mitochondrial dysfunction following injury. However, a critical distinction lies in the regenerative outcome: zebrafish possess a robust capacity to replace damaged hair cells, whereas mammals do not. In the mammalian cochlea, ototoxic damage disrupts mitophagy (e.g., by inhibiting PTEN-induced putative kinase 1 (Pink1)), and restoring mitochondrial health promotes cellular repair (Zhang Y. et al., 2023), indicating that mitochondrial integrity is important for hair cell survival. Nevertheless, whether the mammalian cochlear environment imposes unique metabolic constraints that limit regeneration remains unclear. What is evident is that any attempt at regeneration would require substantial biosynthetic and energetic support, and it has not yet been demonstrated that the adult mammalian cochlea can reliably provide such support without causing additional cellular stress. Finally, the mammalian cochlea possesses a precise tonotopic organization and highly specialized functional division between inner and outer hair cells. Any newly generated hair cell must not only differentiate correctly but also localize to the right position, establish proper polarity, form functional connections with appropriate afferent/efferent nerve terminals, and integrate into the complex cochlear amplifier system. This complexity of functional integration establishes an ultimate checkpoint for regeneration, setting a bar far higher than in other species.

In summary, the loss of regenerative capacity for auditory hair cells in mammals is the result of a systemic blockade. This loss of regenerative capacity does not arise from a single defect but rather from multiple layers of synergistically acting restrictions that have gradually evolved, involving cell cycle regulation, signal transduction, epigenetics, extracellular matrix, vascular and metabolic factors, as well as neural integration. Collectively, these checkpoints ensure extreme stability and longevity of the cochlea in adulthood, but they also permanently seal off the regenerative pathway. Therefore, the core concept of future therapeutic strategies for hair cell regeneration should focus on identifying safe, controllable, and orderly approaches to relieve or bypass the above-mentioned multiple inhibitory checkpoints, thereby reactivating the dormant ancient regenerative programs in the genome, without inducing tumorigenesis or compromising preexisting auditory function.

The propositions of these three checkpoints are grounded in accumulating experimental evidence across multiple species. To provide a clear and systematic overview of the key findings supporting this hypothesis, we summarize the representative studies from various animal models and their relevance to each checkpoint in Table 1.

**TABLE 1:** Representative experimental evidence supporting the multiple checkpoints hypothesis.

Checkpoint	Species	References	Relevance to the hypothesis
Checkpoint one: deep quiescence and epigenetic locking of progenitor cells	Mouse	Kolla et al. (2020)	High-resolution single-cell atlas of mouse cochlear epithelium across developmental stages reveals the progressive restriction of supporting cell plasticity and the establishment of quiescence-associated transcriptional programs after birth, providing a developmental baseline for understanding the deep quiescence described in checkpoint one
	Mouse	Cheng et al. (2019)	Provides transcriptomic evidence for age-related molecular changes in supporting cells, supporting the establishment of quiescence-associated programs
	Chicken, Mouse	Wu et al. (2023)	Cross-species single-cell comparison reveals that mammalian cochlear supporting cells exhibit a more closed chromatin state and downregulation of regeneration-associated gene modules compared to avian species, providing direct evidence for the epigenetic locking mechanism underlying checkpoint one
	Mouse	Tang et al. (2022)	Demonstrates that the epigenetic regulator Dnmt1 controls cell cycle progression and Fgf signaling, linking chromatin regulation to both proliferative quiescence and signal responsiveness
Checkpoint two: silencing of pro-regenerative signaling pathways	Mouse	Li et al. (2015)	Notch inhibition promotes hair cell regeneration by activating the Wnt pathway, revealing functional crosstalk between signaling cascades and demonstrating that regenerative signaling can be pharmacologically engaged
	Zebrafish	Lush et al. (2019)	Single-cell transcriptomic profiling of the zebrafish lateral line during regeneration identifies distinct supporting cell populations that rapidly activate Wnt and Fgf signaling upon hair cell ablation, demonstrating the dynamic pro-regenerative signaling response that mammals lack
	Multispecies vertebrates	Wang et al. (2024)	Cross-vertebrate single-cell atlas comparison reveals that pro-regenerative signaling pathway activity (e.g., Wnt, Fgf) is significantly reduced in mature mammalian cochlea compared to non-mammalian species, directly supporting the characterization of checkpoint two as a silencing of endogenous regenerative signals
Checkpoint three: microenvironmental deterioration	Mouse	Zhang Y. et al. (2023)	Highlights the critical role of metabolic homeostasis (mitophagy) in supporting hair cell survival and regeneration, demonstrating that metabolic dysfunction constitutes a key microenvironmental barrier
	Mouse	Patel et al. (2024)	Demonstrates that cochlear vascular pathology directly leads to hair cell loss and that restoring vascular integrity prevents degeneration, establishing vascular support as a critical component of the permissive microenvironment
	Zebrafish	Barthelaix et al. (2025)	Implicates macrophage functional state as a key modulator of regeneration, revealing that immune microenvironment dysregulation contributes to regenerative decline
	Chicken	Zhang J. et al. (2023)	Demonstrates that decellularized extracellular matrix scaffolds from regenerative species support hair cell differentiation, indicating that ECM composition is a critical determinant of microenvironment permissiveness
Cross-species validation and synergistic mechanisms	Zebrafish	Jimenez et al. (2022)	Demonstrates the “enhanced network” (Sox/Six transcription factors) that enables efficient regeneration in a regeneration-competent species, illustrating the species-specific regulatory modifications superimposed on the core regenerative module
	Chick	Janesick et al. (2022)	Reveals JAK/STAT-dependent immune-related gene expression in supporting cells during avian regeneration, supporting the concept that immune modulation represents a species-specific regulatory modification
​	Chick	Sato et al. (2024)	Reveals the specialized “repair mode” of avian regeneration, distinct from developmental recapitulation, highlighting the complexity of functional integration required for successful regeneration
	Multispecies vertebrates	Wang et al. (2024)	Cross-vertebrate single-cell atlas comparison demonstrates that core sensory cell types are conserved, while regenerative capacity divergence correlates with species-specific transcriptional states and signaling pathway activation patterns, supporting the hypothesis that multiple checkpoints collectively lock regeneration in mammals and that single-pathway intervention is likely insufficient

The table summarizes key findings from diverse experimental approaches, including single-cell transcriptomics (Kolla et al., 2020; Jimenez et al., 2022), bulk RNA-seq (Cheng et al., 2019), genetic lineage tracing (Li et al., 2015), and functional studies (Patel et al., 2024; Arkenberg et al., 2025), collectively supporting the proposed checkpoint framework.

#### 2.3.4 Checkpoint synergy: from independent barriers to a self-reinforcing inhibitory network

The three checkpoints described above do not act in isolation to block regeneration; rather, they have evolved in mammals into an interconnected, self-reinforcing inhibitory network. First, a metabolic-epigenetic coupling exists between Checkpoint 1 and Checkpoint 3: the deep quiescent state of supporting cells is accompanied by downregulation of mitochondrial respiration, and this low metabolic state further reinforces G1/S phase arrest by stabilizing cell cycle inhibitors (e.g., p27^Kip1) and maintaining a closed chromatin conformation (e.g., enrichment of H3K9me3) (Liu et al., 2024). Second, Checkpoint 2 and Checkpoint 1 mutually reinforce each other, forming a bidirectional inhibitory loop. On one hand, the absence of pro-regenerative signals such as Wnt/β-catenin not only directly reduces transcription of cell cycle-related genes but also impairs recruitment of chromatin remodeling complexes to the promoter regions of proliferation-associated genes, contributing to progressive chromatin closure (Pascual-Carreras et al., 2023). On the other hand, emerging evidence indicates that epigenetic changes can precede and predispose the loss of signaling responsiveness in the mammalian cochlea. Tao et al. (2022) demonstrated that epigenetic silencing of pro-regenerative genes occurs prior to the loss of Notch responsiveness, and Shi et al. (2024) showed a progressive loss of chromatin accessibility at regeneration-associated loci in mammals compared to regenerative species. Thus, regardless of whether the initial insult is signal loss or epigenetic silencing, the consequence is a self-reinforcing state of “chromatin closure and blunted signal responsiveness” that stabilizes regenerative quiescence in mammals. Finally, Checkpoint 3 suppresses Checkpoints 1 and 2 through paracrine mechanisms: persistently elevated inflammatory factors (e.g., TGF-β) in the post-injury microenvironment upregulate p27^Kip1 expression while simultaneously inhibiting Wnt ligand secretion (Peng et al., 2022), tightly coupling microenvironmental deterioration with intrinsic regenerative quiescence. Thus, regenerative failure in the mammalian cochlea does not represent a simple summation of three independent defects, but rather a highly coupled, systemic state of lockdown. This insight carries fundamental implications for therapeutic strategies: interventions targeting a single checkpoint are unlikely to break this closed loop, and any successful regenerative approach must coordinately dismantle these three interlocked barriers.

## 3 Breaking the multiple checkpoints: from mechanistic insights to combinatorial intervention strategies

Based on the “Multiple Checkpoints” hypothesis and the synergistic locking mechanism described in Section 2.3.4, regenerative failure in the mammalian cochlea arises from a coupled inhibitory network formed by three interdependent checkpoints. This understanding carries fundamental implications for the design of therapeutic strategies: any attempt to activate regeneration through a single pathway will be thwarted by the persistent presence of the other checkpoints. From this perspective, this chapter systematically reviews the evolution of existing intervention strategies. Section 3.1 focuses on attempts at single-target interventions and their limitations, revealing how relief of one checkpoint leads to new barriers imposed by the remaining checkpoints. Section 3.2 examines efforts aimed at microenvironmental remodeling, which, despite improving local conditions, fail to address the intrinsic regenerative quiescence of supporting cells. Section 3.3 uses stem cell therapy as a case study to illustrate the systemic challenges faced by exogenous cell replacement strategies. Synthesizing these analyses, we propose that future regenerative strategies must shift from “single-point breakthrough” to “combinatorial intervention,” simultaneously targeting the three interlocked checkpoints—intrinsic reprogramming barriers, silencing of pro-regenerative signaling pathways, and microenvironmental deterioration—to truly awaken the dormant regenerative potential within the mammalian cochlea.

### 3.1 Attempts and challenges in overcoming cell cycle blockade

Overcoming “Checkpoint One” by reactivating quiescent supporting cells to re-enter the cell cycle is the primary step toward achieving regeneration. It is known that cell cycle inhibitory proteins such as p27Kip1 and pRB constitute a robust G1/S-phase barrier (Laos et al., 2014; Walters et al., 2014). However, studies indicate that merely relieving these intrinsic inhibitions alone is insufficient to drive an efficient and safe regenerative program. Early intervention strategies focused on directly modulating the cell cycle engine. For instance, overexpression of G1-phase cyclins (e.g., Cyclin D1) or silencing of cyclin-dependent kinase inhibitors (e.g., p27) via gene knockout *in vitro* or *in vivo* models (Deshmukh et al., 2022) can, to a limited extent, promote the re-entry of supporting cells into S-phase. Yet, these approaches are often inefficient and may induce unspecific proliferation or apoptosis, highlighting the limitations and potential risks of cell-autonomous cell cycle reactivation.

Successful cell cycle reactivation is not an isolated event; it requires coordination with exogenous pro-mitogenic signals. This directly addresses the core issue of the silencing of endogenous pro-regenerative signaling pathways, which constitutes “Checkpoint Two.” Research has confirmed that downregulation of the epigenetic regulator DNA methyltransferase 1 (Dnmt1) can disrupt the normal balance between proliferation and differentiation by affecting Wnt and FGF signaling pathways (Tang et al., 2022). This finding profoundly reveals that dismantling the intrinsic cell cycle brakes must be accompanied by the concurrent provision of exogenous “permissive signals,” such as Wnt and FGF, to mimic the post-injury microenvironment of regenerating animals. However, achieving precise temporal, spatial, and dosage-controlled delivery and regulation of these signals within the complex and inhibitory environment of the adult human cochlea remains a significant challenge in current research.

In summary, an effective strategy for overcoming the cell cycle checkpoint must focus on the temporally coordinated regulation of intrinsic inhibition relief and exogenous permissive signal activation. Future research needs to develop intelligent delivery systems or genetic circuits that can precisely replicate this synergistic interaction at the site of injury, thereby safely and controllably reactivating the proliferative potential of supporting cells (Schimmang and Pirvola, 2013).

### 3.2 Microenvironment remodeling: strategies for transforming from an inhibitory to a permissive state

The success of regeneration depends not only on the reactivation of the cells themselves but also on reprogramming their surrounding inhibitory homeostatic microenvironment into a permissive state. This lies at the core of overcoming “Checkpoint Three.” Current strategies aim to systematically remodel the cochlear microenvironment across multiple dimensions.

First, nutritional and vascular support are fundamental for maintaining cellular health and regenerative activity. Studies indicate that zinc deficiency triggers abnormal expression of cell cycle arrest-related proteins within the cochlea, while zinc supplementation reverses this effect, revealing that specific nutrient metabolites are critical microenvironmental elements for preserving the proliferative potential of inner ear cells (Yi et al., 2022). More importantly, the integrity of the cochlear vascular system directly dictates hair cell fate. For instance, in Norrie disease protein (Ndp) gene-knockout mice, restoring cochlear vascular function effectively prevents progressive hair cell loss (Patel et al., 2024). These studies provide strong evidence that a metabolically supportive microenvironment, sustained by a healthy vascular network and adequate nutrition, is a prerequisite for any regenerative attempt to proceed.

Secondly, the active modification of the physical and chemical microenvironment has emerged as a Frontier direction. Utilizing biomimetic extracellular matrix (ECM) hydrogels or biomaterials enables the construction of three-dimensional scaffolds that support hair cell differentiation and neuronal synapse growth. These artificial ECMs not only provide structural support but can also serve as sustained-release carriers for growth factors (e.g., BDNF, NT-3), creating a localized, sustained, and controllable permissive signaling environment (Arkenberg et al., 2025; Zhang J. et al., 2023). After injury, the polarization state of immune cells such as macrophages—specifically the transition from the pro-inflammatory M1 phenotype to the reparative M2 phenotype—exerts a profound influence on the resolution of inflammation and the process of tissue repair (Barthelaix et al., 2025; Du et al., 2024; Martin and García, 2021). This provides new avenues for promoting regeneration through immune modulation. It should be noted, however, that the requirement for immune responses in hair cell regeneration is not universal across vertebrates; in zebrafish, successful hair cell regeneration can proceed without adaptive immune responses (Warchol et al., 2020) This suggests that the contribution of immune modulation to regeneration is likely species- and context-dependent. Therefore, the core concept of future therapeutic strategies for hair cell regeneration should focus on identifying safe, controllable, and orderly approaches to relieve or bypass the above-mentioned multiple inhibitory checkpoints, thereby reactivating the dormant ancient regenerative programs in the genome, without inducing tumorigenesis or compromising preexisting auditory function.

In summary, microenvironmental remodeling is a multi-target, systemic endeavor. Future therapeutic design must move beyond supplementing individual signaling molecules and instead pursue the integration of vascularization, nutrient delivery, immune modulation, and biophysical scaffolds. The goal is to construct a comprehensive, regeneration-compatible system that holistically supports hair cell regeneration, survival, and functional integration.

### 3.3 Challenges in inner ear hair cell regeneration: a case study using stem cells

When the regenerative potential of endogenous supporting cells is deemed exceptionally difficult to reactivate, exogenous stem cell transplantation or the activation of endogenous stem/precursor cells becomes the most attractive alternative strategy. However, the ultimate success of this alternative pathway depends on its ability to overcome subsequent microenvironmental and integration checkpoints.

Stem cell therapy holds immense theoretical potential. Research has confirmed that inner ear progenitor cells capable of proliferation and hair cell differentiation can be isolated from early-stage cochlear or vestibular tissues (Oshima et al., 2007). Even more remarkably, through precise temporal signaling induction of pluripotent stem cells (e.g., embryonic stem cells or induced pluripotent stem cells), scientists have successfully generated cells *in vitro* that exhibit structural, transcriptomic, and functional characteristics of both inner and outer hair cells, with a maturation level comparable to late developmental stages (Koehler et al., 2017; Menendez et al., 2020). This provides a promising avenue for obtaining a substantial source of regenerative cells.

However, successfully integrating these cells into the adult mammalian cochlea to restore function faces fundamental challenges that are direct manifestations of the “Multiple Checkpoints” hypothesis. First, the survival, migration, and engraftment of transplanted cells are directly constrained by the inhibitory microenvironment (Checkpoint Three) of the adult cochlea. The lack of vascular support and the presence of an inhibitory ECM are detrimental to the survival of exogenous cells (Park et al., 2025; Xu et al., 2025). Second, functional differentiation and neural connectivity of the cells require precise spatiotemporal signal guidance, which involves the exact regulation of signals such as Wnt and Notch (Checkpoint Two) (Samarajeewa et al., 2019). Ultimately, the challenge of functional integration—forming specific and stable synaptic connections with host spiral ganglion neurons—must be addressed. Currently, the differentiation efficiency, positional specificity, and innervation rate of transplanted cells *in vivo* remain far from meeting the requirements for functional recovery.

Therefore, stem cell therapy is not a standalone solution; its success critically depends on deep integration with the aforementioned microenvironmental remodeling strategies. The future direction lies in constructing an integrated “cell-material-signal” composite therapeutic system. This involves co-delivering stem or progenitor cells to the cochlea alongside biomaterials carrying angiogenic factors, neurotrophic factors, and immunomodulatory molecules. This approach actively remodels the transplantation site microenvironment, collectively creating a “regenerative niche” that enables cell survival, directed differentiation, functional maturation, and integration with the host neural circuitry.

In summary, the three major challenges facing stem cell therapy—survival, differentiation, and integration—directly reflect the three checkpoints posited by the “Multiple Checkpoints” hypothesis: the difficulty of transplanted cells surviving in an inhibitory microenvironment corresponds to Checkpoint 3 (microenvironmental deterioration); the requirement for directed differentiation signals corresponds to Checkpoint 2 (silencing of pro-regenerative signaling pathways); and functional integration with host neurons involves the coordinated relief of the first two checkpoints. This analysis further reinforces a central tenet of this chapter: whether activating endogenous supporting cells (Section 3.1) or transplanting exogenous stem cells, interventions targeting a single dimension are unlikely to achieve success. A successful regenerative strategy must evolve toward an integrated “cell-material-signal” combinatorial therapeutic system, a concept that will be further elaborated in the following chapter.

## 4 Discussion

The “Multiple Checkpoints” hypothesis proposed in this study offers a conceptual framework for understanding the evolutionary loss of auditory hair cell regenerative capacity in mammals. Here, we discuss the implications of this model, its alignment with existing evidence, and its distinctions from current paradigms.

### 4.1 Evolutionary trade-offs and regenerative capacity

The hypothesis posits that regenerative failure in mammals represents an evolutionary trade-off, in which structural stability and specialized auditory function are prioritized over cellular plasticity. The progressive strengthening of regenerative barriers across multiple levels parallels the increased structural and functional complexity of the mammalian cochlea. We propose that the extreme cytoarchitectural precision required for mammalian-specific auditory specializations—including the outer hair cell-driven cochlear amplifier, sharp tonotopic organization, and highly stable synaptic connectivity—has suppressed plastic and regenerative responses that would otherwise disrupt the delicate architecture of the organ of Corti.

Notably, this trade-off depends on cellular and structural specializations rather than simply hearing frequency range. Although birds (including owls) exhibit high-frequency hearing comparable to mammals, their basilar papilla lacks the unique structural complexity of the mammalian cochlea (Jahan et al., 2018). The avian sensory epithelium maintains a relatively simple, uniform cytoarchitecture in which supporting cells retain proliferative plasticity (Janesick and Heller, 2019). By contrast, the mammalian organ of Corti is highly compartmentalized and rigid, composed of terminally differentiated, functionally specialized cell types whose post-mitotic stability is essential for sensitive sound amplification and frequency tuning. Even in high-frequency hearing specialists such as barn owls, the simpler structure of the basilar papilla allows regeneration without impairing auditory function whereas the fragile, highly ordered mammalian cochlea cannot tolerate proliferation-associated disruption.

This evolutionary trade-off thus accounts for the broad pattern of regenerative capacity across vertebrates: robust regeneration in anamniotes such as fish and amphibians, moderate regeneration in birds with specialized hearing, and minimal regeneration in mammals, which prioritize structural stability for high-precision auditory function. The suppression of regeneration is therefore systemic, involving multiple synergistic constraints rather than a single genetic switch. This framework shifts therapeutic target discovery from a reductionist search for a “master switch” toward a systems-level understanding of regenerative regulation.

### 4.2 Reconciling clinical observations with the model

A key strength of this framework is its explanatory power for clinical data. Recent reports of transient hearing improvement following anti-inflammatory (Natarajan et al., 2023), antioxidant (Kitama et al., 2025), and microcirculation-enhancing therapies have often been interpreted with cautious optimism. Our model provides a mechanistic explanation for these observations: such interventions primarily ameliorate microenvironmental checkpoints, reducing stress and creating conditions conducive to the survival and repair of existing hair cells or synapses (Ahmadzai et al., 2019; Zhu et al., 2023). However, they do not address the intrinsic checkpoints within supporting cells (e.g., epigenetic silencing, lack of progenitor activation). This explains why the observed functional recovery is typically partial and temporary rather than a true restoration of auditory function through *de novo* hair cell generation. The clinical data thus validate the hierarchical prediction of our hypothesis: addressing individual checkpoints yields repair, while regeneration demands coordinated, multi-target intervention.

### 4.3 Implications for future research directions

If regenerative failure is indeed a multi-layered problem, then therapeutic strategies must evolve from single-target approaches toward synergistic combinations. Three critical challenges emerge from this framework, and addressing them will be essential for translating the hypothesis into therapeutic reality.

First, mechanistic validation requires models that can recapitulate the complexity of the inner ear. Organoid systems offer a platform to test whether simultaneously targeting multiple nodes—for instance, combining Notch inhibition with pro-regenerative factor overexpression—can overcome the synergistic barriers predicted by the model. A key question is whether newly generated cells in such systems can achieve functional integration or remain constrained by microenvironmental factors. Second, therapeutic innovation must prioritize safety and precision. Unlocking the regenerative program without causing dysplastic or neoplastic growth necessitates novel tools capable of temporal, spatial, and dosage control. Advances in gene editing (Wang and Puel, 2018; Zhu et al., 2024) and stem cell biology (Tao et al., 2022) provide the foundational insights, but their translation into clinical therapies will depend on delivery systems that can navigate the delicate inner ear environment. Third, the field requires refined evaluation paradigms. As our hypothesis emphasizes the distinction between repair and regeneration, future studies must use high-resolution imaging, specific biomarkers, and synaptic function analysis to rigorously differentiate these outcomes. Without such granular assessment, partial functional improvements may be misinterpreted as regenerative success, underscoring the need for a paradigm shift toward mechanistically informed therapeutic strategies.

## 5 Conclusion

We propose the “Multiple Checkpoints” hypothesis to explain the evolutionary loss of auditory hair cell regeneration in mammals. Regenerative failure arises not from absence of core genetic programs, but from superimposition of multiple inhibitory barriers at key nodes: progenitor activation, signaling initiation, and microenvironmental support. This framework conceptualizes regenerative failure as an evolutionary trade-off, where plasticity is sacrificed for cochlear structural precision. Synergistic checkpoints explain why partial interventions yield transient functional improvements rather than true regeneration. The hypothesis provides mechanistic explanation for clinical observations: anti-inflammatory and antioxidant interventions ameliorate microenvironmental checkpoints, facilitating repair of existing cells but failing to initiate *de novo* hair cell generation. A fundamental question is why mammals lost regenerative capacity while anamniotes retained it. Reframing regenerative failure as a systemic, multi-layered problem shifts the paradigm from searching for single “master switches” toward understanding synergistic barrier interactions.

These findings point to three future directions: mechanistic validation using organoid and other models, development of precision delivery tools, and establishment of evaluation paradigms that distinguish repair from genuine regeneration. Addressing these challenges will be essential for translating the Multiple Checkpoints hypothesis into therapeutic reality.
